# Visual Loss in Postconflict Southern Sudan

**DOI:** 10.1371/journal.pmed.0030450

**Published:** 2006-12-19

**Authors:** John Buchan

## Abstract

Buchan discusses two studies in southern Sudan that provide the first estimates of the prevalence and causes of blindness and low vision for this region since before the 21-year conflict began.

Recent global estimates of the number of blind people put the total at 37–42 million [1,2]. This figure is predicted to rise to over 75 million by the year 2020 as a result of increases in world population and life expectancy, if current age-specific rates of blindness are allowed to persist [3].

The distribution of this global burden is, however, far from even. Blindness disproportionately affects the socioeconomically disadvantaged, both in terms of the numbers affected and in the impact of blindness on quality and length of life [4]. Although this link between blindness and low socioeconomic status is well known, two new studies published in *PLoS Medicine* are a useful reminder of just how vulnerable the poorest communities are to blindness [5,6].

## The New Studies

The two studies by Jeremiah Ngondi and colleagues were conducted in the Mankien district of southern Sudan. The studies provide the first estimates of the prevalence and causes of blindness and low vision for this region since before the 21-year conflict began. They also provide new data on the prevalence of blinding trachoma.

The reasons for undertaking such surveys are well known. The data produced provide the basis from which to (1) plan the magnitude and direction of eye health-care services; (2) define priority areas for interventions; (3) set targets and monitor progress towards those targets; and (4) permit informed advocacy and highlight areas where further needs assessment is required [7]. These timely surveys by Ngondi et al. will prove valuable, therefore, only as long as the existence of peace in the region and availability of resources allow implementation of targeted interventions.

From the 61 villages in the Mankien district that were deemed accessible, a random sample of 22 villages was taken; 25 households from each village were then selected and the consenting occupants examined. Overall prevalence of blindness (presenting visual acuity less than 3/60 in the better eye) amongst those over five years of age was estimated at 4.1%, with a further 7.7% having low vision (presenting visual acuity less than 6/18 and equal to or better than 3/60 in the better eye). This compares to the threshold blindness prevalence of 0.5% (whole population) for a subregion, or 1% for any community, set by the World Health Organization as the maximum acceptable [8]. Most remarkably, in those aged over 50 years, almost one quarter were recorded as blind and more than half of the remainder were found to have low vision.

Blindness disproportionately affects the socioeconomically disadvantaged.

The possibility of over-estimation due to selection bias is discussed in an accompanying Perspective [9]. Nevertheless, the impact that visual impairment, at anything approaching this scale, would have on a community should not be forgotten. Productivity is lost, not only from the individual affected, but also from the family member removed from active work or education in order to provide the informal care required.

## The Burden of Trachoma

In their new studies, Ngondi et al. identified cataract as the leading cause of blindness, accounting for around 40% of cases, broadly in keeping with the worldwide figures [1]. By contrast, trachoma was responsible for 35.3% of blindness, 10 times its proportion globally [1]. Of children aged one to nine years, 63.3% were found to have signs of active trachoma, and 19.2% of the population over 15 years of age had trichiasis, compared to the 10% and 1% respective World Health Organization thresholds for declaring trachoma a public health problem [7].

In the setting of a health-care system in an industrialised country, with around one ophthalmologist per 20,000 population, the identification of a single case of active trachoma might prompt an ophthalmologist to be flown in to screen the entire community and provide mass distribution of antibiotics [10]. However, where there is less than one ophthalmologist per million population [11], a coordinated public health intervention involving non-doctor surgeons is required, as embodied in the SAFE strategy (Surgery, Antibiotics, Facial Cleanliness, Environmental Change).

Vision 2020: The Right to Sight (http://www.v2020.org), the international collaborative initiative to eliminate avoidable blindness by the year 2020, has named trachoma as one of its five target diseases. Trachoma has indeed been observed to decline markedly in many countries, both as a result of coordinated disease control efforts and of improved sanitation and standards of living [12]. The surveys by Ngondi et al. remind us that whilst trachoma may be decreasing globally, it is alive and well in the villages of the most disadvantaged. It continues to blind, and will continue to do so for generations to come if nothing is done to interrupt the cycle of infection and re-infection as children with sticky eyes grow into irreversibly blind adults [13].

## Conclusion

The tragedy of blindness due to trachomatous corneal scarring is that it is not curable. By contrast, the tragedy of cataract blindness is that it is eminently curable. The consumables for cataract surgery, including an implantable intra-ocular lens, are available for less than US$10 in a developing world setting [14]. Ngondi et al. present for us a picture of the burden of disease that is being carried by a people already burdened by the legacy of two decades of war. The challenge for health-care providers is to find the resources required to offer treatment for treatable causes of blindness and to prevent that which can not be treated.
